# Dataset for mosquito collections on Big Pine Key, Florida, USA

**DOI:** 10.1016/j.dib.2019.104516

**Published:** 2019-09-14

**Authors:** Lawrence J. Hribar

**Affiliations:** Florida Keys Mosquito Control District, 503 107th Street, Marathon, FL, 33050, USA

**Keywords:** Culicidae, Mosquito, Seasonal distribution, Relative abundance, *Aedes*, *Anopheles*, *Culex*, *Deinocerites*, *Psorophora*, *Uranotaenia*, *Wyeomyia*

## Abstract

The Florida Keys Mosquito Control District has deployed dry ice-baited light traps to monitor mosquito (Diptera: Culicidae) populations throughout the Florida Keys starting in 1998. American Biophysics Company traps were deployed throughout the year at the same collection point. Traps were placed in the late afternoon and collected the following morning. Common mosquitoes are the black salt marsh mosquito, *Aedes taeniorhynchus* (Wiedemann), the crabhole mosquito, *Deinocerites cancer* Theobald, the Bahamian Culex*, Culex bahamensis* Dyar and Knab, and *Anopheles atropos* Dyar and Knab.

**Table:** Specifications Table

Subject	Insect Science
Specific subject area	Entomology, Seasonal Distribution, Relative Abundance
Type of data	Spreadsheet
How data were acquired	Mosquito collections on Big Pine Key, Florida, USA
Data format	Raw
Parameters for data collection	Mosquitoes were counted and identified in the laboratory.
Description of data collection	Dry ice-baited light traps were deployed to monitor mosquito (Diptera: Culicidae) populations on Big Pine Key Keys starting in 1998. American Biophysics Company traps were deployed throughout the year at the same collection point. Traps were placed in the late afternoon and collected the following morning.
Data source location	Big Pine Key, Monroe County, Florida United States
Data accessibility	With the article

**Table:** 

**Value of the Data** • The data provide a twenty-year sample of mosquito populations on one of the Florida Keys • The data may benefit persons studying seasonal distribution, relative abundance, species composition, and other aspects of mosquito biology in southern Florida. • The data may prove to be of value to persons researching changes in populations or communities over time especially in relation to climate, land use, or other factors.

## 1 Data

The data are in an Excel spreadsheet. The spreadsheet contains 999 rows, 1 for column headers and 998 for data. There are 39 columns, the first for the year of collection, the second for the day of the year of collection, and the remainder for mosquito species collected. Column labels are as follows: YEAR, year of collection; DOY, day of year; TAEN, *Aedes taeniorhynchus*; DEIN, *Deinocerites cancer*; NIGR*, Culex nigripalpus*; ATRO, *Anopheles atropos*; QUIN, *Culex quinquefasciatus*; CRUC, *Anopheles crucians*; BAHA, *Culex bahamensis*; INFI, *Aedes infirmatus*; ATLA, *Aedes atlanticus*; CILI, *Psorophora ciliata*; COLU, *Psorophora columbiae*; SOLL, *Aedes sollicitans*; AEGY, *Aedes aegypti*; TRIS, *Aedes triseriatus*; LOWI, *Uranotaenia lowii*; QUAD, *Anopheles quadrimaculatus*; ERRA, *Culex erraticus*; ATRA, *Culex atratus*; IODO, *Culex iodolambdis*; PECC, Culex peccator; UNIM, unidentified *Culex* (*Melanoconion*) sp.; SALI, *Culex salinarius*; MELA, *Culiseta melanura*; WYMI, *Wyeomyia mitchelli*, FERO, *Psorophora ferox*; JOHN, *Psorophora johnstonii*, TORT, *Aedes tortillis*; ALBI, *Anopheles albimanus*; COND, *Aedes condolescens*; MULR, *Culex mulrennani*, PILO, *Culex pilosus*; DECL, *Culex declarator*; INOR, *Culiseta inornata*; GRAB, *Anopheles grabhamii*; TITI, *Mansonia tittilans*; UNID, unidentified; TOTAL, total mosquitoes collected that day.

## 2 Experimental design, materials, and methods

Mosquitoes were collected at the northern end of Key Deer Boulevard on Big Pine Key. Surrounding vegetation includes black mangroves (*Avicennia germinans* L.), white mangroves (*Laguncularia racemosa* (L.) Gaetern. f.), red mangroves (*Rhizophora mangle* L.), buttonwood (*Conocarpus erecta* L.), saltwort (*Batis maritima* L.), and other halotolerant plants typical of the Florida Keys [1], [2], [3]. Battery-powered, carbon dioxide-baited light traps were placed in the field weekly from 1998 until 2019. Traps were deployed in the late afternoon and retrieved the following morning. Mosquitoes collected were transported to the laboratory, killed by freezing, and identified to species and counted [3]. The intent was to make collections on a weekly basis but due to storms, illness, and other events this was not always possible.
